# Giant retroperitoneal liposarcoma

**DOI:** 10.1186/1477-7819-6-115

**Published:** 2008-10-31

**Authors:** Ángel Herrera-Gómez, César Ortega-Gutiérrez, Alejandro Mohar Betancourt, Kuauhyama Luna-Ortiz

**Affiliations:** 1 Department of Surgical Oncology, Instituto Nacional de Cancerología, México, D.F., Mexico; 2 Department of Pathology, Instituto Nacional de Cancerología, México, D.F., Mexico

## Abstract

**Background:**

Liposarcoma is the most frequent histopathological variety of the retroperitoneum, surgery is the gold standard for treatment.

**Case presentation:**

We present the case of a 24-year-old male who was diagnosed with a giant retroperitoneal liposarcoma. The patient received palliative treatment due to non-resectability on the basis of chemotherapy. We decided to perform surgery after no benefit was received with systemic treatment. Complete macroscopic resection of the tumor was performed, without multi-organ resection. The patient is currently alive and disease free at 14 months of evolution.

**Conclusion:**

Retroperitoneal liposarcomas represent a unique situation and require a more aggressive surgical approach including multiple resections for recurrences. Based on the ability of the patient to tolerate the procedure, surgery is suggested to evaluate resectability of the tumor. We must take into consideration whether prolonged survival will be attained and tumor removal will result in palliation of symptoms.

## Background

Liposarcomas are neoplasms of mesodermic origin derived from adipose tissue and correspond to 10–14% of all soft tissue sarcomas. They represent < 1% of all malignant tumors [1,2].

The most frequent subtypes are liposarcoma (41%), leiomyosarcoma (28%), malignant fibrous histiocytoma (7%), fibrosarcoma (6%) and tumors of the peripheral nerve sheath (3%) [3]. Retroperitoneal liposarcomas alone comprise 0.07–0.2% of all neoplasias^4^. Approximately 85% of these are malignant, with soft-tissue sarcomas representing 35% of this group. Liposarcoma is the most frequent histopathological variety of the retroperitoneum [2]. It presents with inherent characteristics in relation to its deep localization and slow expansive growth. Average diameter of the tumor is 20–25 cm with a weight of 15–20 kg [4]. There is compromise of the adjacent organs in up to 80% of the cases [4,5]. Surgery is the gold standard for treatment of liposarcoma. Retroperitoneal liposarcoma is a distinct clinical entity that requires a more aggressive surgical approach, including multiple resections or multiorgan resection with recurrences. There is a low incidence of distance metastasis (7%) compared to other histological subtypes that range from 15 to 34% [6]. The objective of this study is to report a case of giant retroperitoneal sarcoma.

## Case presentation

**Figure 1:**
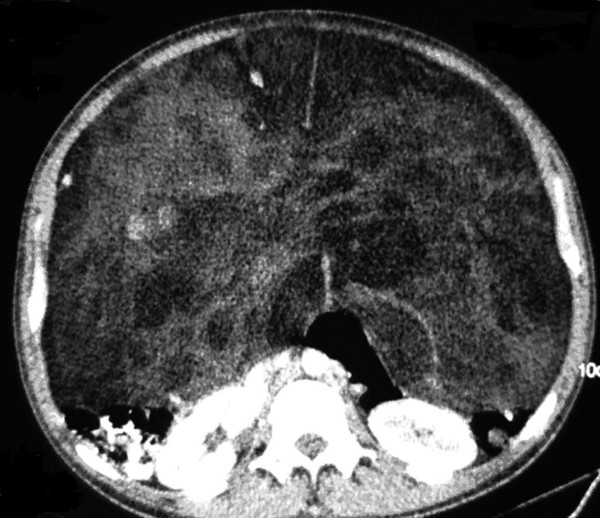
CT scan. Heterogeneous lesion is observed with zones of fat and solid density that entirely occupy the abdominal cavity, displacing retroperitoneal structures dorsally.

**Figure 2:**
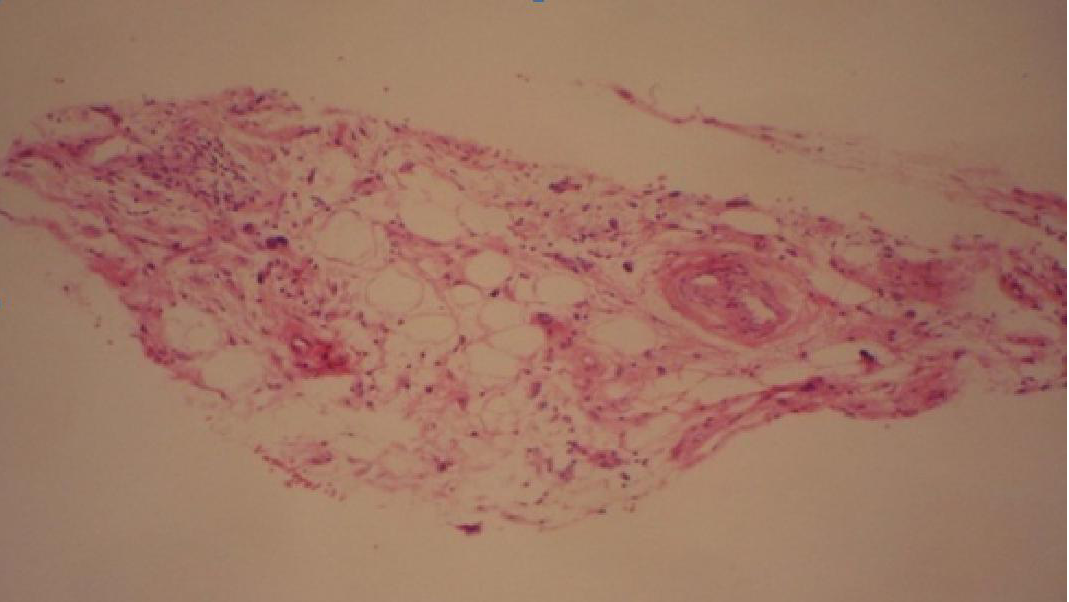
Well-differentiated sclerosing liposarcoma.

**Figure 3:**
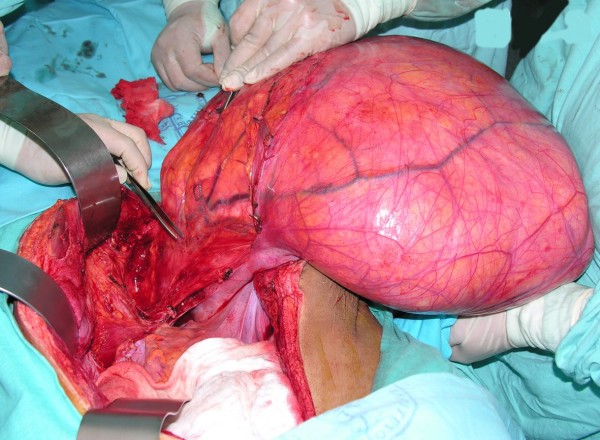
Retroperitoneal tumor (80 × 60 cm), weighing 18 kg. Complete resection.

A 24-year-old male presented with a 6-month evolution of his disease with abdominal pain, constipation, fever and a 20-kg weight loss. He received treatment for typhoid fever without improvement. For this reason, abdominal tomography was performed, demonstrating a heterogeneous lesion with zones of fat and solid density that entirely occupied the abdominal cavity, displacing retroperitoneal structures dorsally. CT-guided biopsy was performed at a different hospital and pathological report demonstrated liposarcoma. Colonoscopy was performed, demonstrating extrinsic compression at the level of the descending colon. The patient was sent to our Institution for further evaluation. Upon admission, the patient had a Karnofsky score of 90 and was classified as grade zero according to the status of the Eastern Cooperative Oncology Group (ECOG). He also presented with respiratory difficulty and distended abdomen due to solid, multilobulated tumor that extended from the epigastrium to the pelvic region without delineated borders. CT scan confirmed previous findings (Fig. 1). Histopathological review from the referring hospital demonstrated minute fragments of mature adipose tissue without atypia. This material was considered to be inadequate for diagnosis. For this reason, a new CT-guided biopsy was performed and a well-differentiated sclerosing-type liposarcoma was reported (Fig. 2). Because the patient's tumor had previously been considered nonresectable, he was referred to medical oncology where chemotherapy with ifosfamide and adriamycin was offered. Clinical and radiological responses were evaluated and reported stable disease. The patient completed six cycles of adriamycin and ifosfamide and was re-evaluated upon treatment completion. The patient continued with stable disease (no change in tumor size was documented either clinically or radiologically) without change in functional status according to Karnofsky index upon admission. At the conclusion of chemotherapy, supportive therapy vs. chemotherapy with etoposide and ifosfamide was proposed and the patient elected supportive therapy. An interdepartmental meeting was held and, in a joint decision with the patient, surgical exploration was decided upon. Surgery was performed with the patient in dorsal decubitus and a midline incision was made from the xyphoid to the pubis, revealing an 80 × 60-cm tumor that encompassed the entire retroperitoneal cavity, with lax adhesions to the descending colon and ureters, neovascularization and adherence to the bladder without multiorgan resection and with macroscopic free margins (Fig. 3). Complete resection of the tumor was performed. The patient had a satisfactory evolution and was discharged 48 h postsurgery without adjuvant treatment. The final histopathological report showed undifferentiated liposarcoma of the retroperitoneum (80 × 50 × 35 cm) weighing 18 kg.

**Figure 4:**
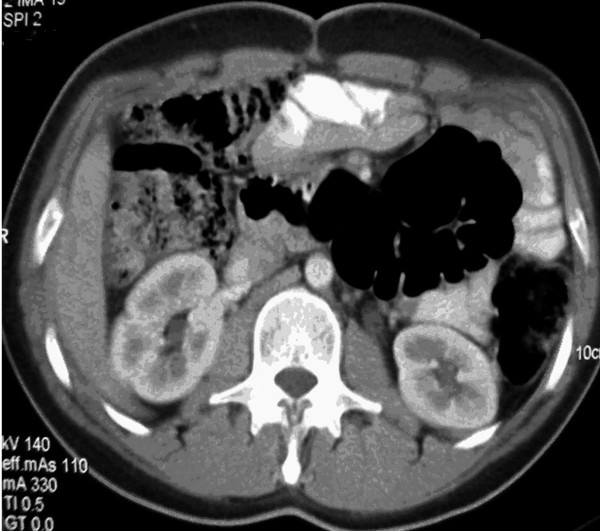
CT scan. No evidence of tumor activity is demonstrated. Localization of intraabdominal organs is adequate.

The patient is being followed-up every 3 months. At 6 months post-surgery, a new CT scan was done and there was no evidence of disease (Fig. 4). Currently, at 14 months of follow-up, the patient is asymptomatic and disease free.

## Discussion

Liposarcoma is the most frequent histological type of retroperitoneal sarcoma, corresponding to 41% of these tumors [4,5]. It has been reported that 20% of the tumors are > 10 cm at the time of diagnosis [3]; however, few cases of retroperitoneal liposarcomas exist that can be considered as giant [4,6-9]. The case we present may be considered among the largest tumors reported for this histology. Clinically, these tumors tend to present with diffuse abdominal pain accompanied by anorexia and weight loss and increase in abdominal girth. The most characteristic sign is a painless abdominal mass detected in ~78% of the cases. Abdominal symptomatology is due to compression of the organs, similar to that reported with the present case [3,4]. It is clear that our patient presented all the signs and symptoms specific for these tumors due to the size of the abdominal mass.

The tumor was initially considered nonresectable and, therefore, systemic treatment with chemotherapy was decided upon, with the goal of reducing tumor load. Various chemotherapy regimens have been described based on mesna, doxorubicin, ifosfamide, dacarbazine and paclitaxel. However, their use is limited for recurrent metastatic disease or palliation. Survival benefits have not been demonstrated [10]. Due to the large tumor load and probable multiorgan involvement, we initially considered palliative treatment for our patient. Because the patient did not demonstrate any beneficial effects from chemotherapy, the joint decision of the hospital committee, along with the patient, was to perform surgery. Complete resection of the tumor was performed. We are in agreement with Patrik et al. [6] who demonstrated that in liposarcomas > 10 cm, complete resection can be carried out in up to 70% of cases; however, in up to 50% of these cases, multiorgan resection is necessary in order to reach this goal [2]. The most frequent organ resected is the kidney (30%). In the case we report here, even with the large size of the tumor, organ resection was not necessary, because there was no infiltration to neighboring structures, only lax adhesions allowing adequate dissection of the tumor, as shown in Fig. 3.

Radiotherapy (RT) was not considered in this case for two principal reasons: the first was due to the large tumor load and no demonstrable reduction in tumor size and, second, because of the probable gastrointestinal morbidity associated with such an extensive field as in this case. With regard to RT as complementary treatment, there is agreement for its palliative use in non-operable tumors or in cases of incomplete resection [7,8]. Although mesodermic tumors are radioresistant, liposarcoma is more radiosensitive [6]. Although it has been noted that RT may increase survival and disease-free interval [3,7,8], other authors reported that this treatment has not demonstrated long-term improvement in survival or specific disease in cases of complete macroscopic resections [6,8]. This occurs despite using intraoperative RT with the goal of increasing efficacy of the local dose with 50–60 Gy [7] and of minimizing toxicity to adjacent organs. In our case, because we were dealing with a well-differentiated primary liposarcoma that allowed complete resection, 5-year survival of 75–100% [9,11] has been described. Local failure occurs within 5 to 10 years after resection in up to 90% of the cases related to size of tumor, inability to achieve free margins and limitations of adjuvant treatment such as chemotherapy or RT [3,4]. We cannot compare these data with our report because our patient is disease free at 14 months of follow-up.

## Conclusion

Retroperitoneal liposarcomas are a unique situation and require a more aggressive surgical approach including, when necessary, multiorgan resection or multiple resections with recurrences. In accordance with the ability of the patient to tolerate the procedure, surgery is suggested to evaluate tumor resectability, taking into consideration prolonged survival. After tumor removal, palliation of symptoms will be accomplished.

## Competing interests

The authors declare that they have no competing interests.

## Authors' contributions

AHG Carried out the surgery procedure, review the manuscript. COG Carried out the surgery and search for literature review and review manuscript. AMB was the pathologist for this case and review the article. KLO Carried out the surgery, write the article, analysis of the literature and review the article. All authors read and approved the final manuscript.

## Consent

Written informed consent was obtained from the patient for publication of this case report.

## References

[B1] Kilkenny JW, Bland KI, Copeland EM (1996). Retroperitoneal Sarcoma. The University of Florida Experience. J Am Coll Surg.

[B2] Hassan I, Park SZ, Donohue JH, Nagorney DM, Kay PA, Nasciemento AG, Schleck CD, Ilstrup DM (2004). Operative management of primary retroperitoneal sarcomas. A reappraisal of an institute experience. Ann Surg.

[B3] Lewis JJ, Leung D, Woodruff JM, Brennan MF (1998). Retroperitoneal soft-tissue sarcoma: analysis of 500 patients treated and followed at a single institution. Ann Surg.

[B4] Echenique-Elizondo M, Amodarain-Arratibel JA (2005). Liposarcoma retroperitoneal gigante. Cir Esp.

[B5] Jaques DP, Coit DG, Hajdu SI, Bennan MF (1990). Management of primary and recurrent soft-tissue sarcoma of the retroperitoneum. Ann Surg.

[B6] McGrath PC, Neifeld, Lawrence W, DeMay RM, Kay S, Horsley JS, Parker DA (1984). Improved survival following complete excision of retroperitoneal sarcomas. Ann Surg.

[B7] Azpiazu Arnaiz P, Muro Bidaurre I, De Frutos Gomero A, Castro Esnal E, Martin Lopez A, Asesnsio Gallego JI, Rivera Garbayo JR (2000). Tumores retroperitoneales. Liposarcoma mixoide retroperitoneal. Presentación de un nuevo caso. Arch Esp de Urol.

[B8] Romero Pérez P, Rafie Mazketli W, Amat Cecilia M, Merenciano Cortina FJ, Gonzalez Devesa M (1996). Tumores adiposos retroperitoneales. A propósito de un liposarcoma mixoide gigante. Actas Urol Esp.

[B9] Guzman Martinez-Valls Pl, Ferrero Doria R, López Alba J, Tomas Ros M, Rodenas Moncfada FJ, Rico Galiano JL, Rodríguez de Ledesma Vega JM, Fontana Compiano LO (1997). Liposarcoma retroperitoneal. A propósito de tres casos. Arch Esp de Urol.

[B10] Yoshida Y, Inoue K, Ohsaco T, Nagamoto N, Tanaka E, Tsuruzoe S (2007). Weekly paclitaxel therapy is curative for patients with retroperitoneal liposarcoma. Gan To Kagaku Ryoho.

[B11] Mehrotra PK, Ramachandran CS, Goel D, Arora V (2006). Inflammatory variant of a well-differentiated retroperitoneal liposarcoma: case report of a rare giant variety. Indian J Cancer.

